# History of the administration of psychedelics in France

**DOI:** 10.3389/fpsyg.2023.1131565

**Published:** 2023-09-01

**Authors:** Zoe Dubus, Elise Grandgeorge, Vincent Verroust

**Affiliations:** ^1^Laboratoire TELEMMe, Faculté des Arts, Lettres, Langues, Sciences Humaines, Aix-Marseille Université, Marseille, France; ^2^Centre Hospitalier Universitaire Vaudois (CHUV), Institut des Humanités en Médecine, Lausanne, Switzerland; ^3^Psychiatrie-Comorbidités-Addictions (PSYCOMadd), Université Paris-Sud, Paris, France; ^4^Histoire des arts et des Représentations, Université Paris Nanterre, Nanterre, France; ^5^Centre d'histoire des Sociétés, Des Sciences et des Conflits, University of Picardie Jules Verne, Amiens, France

**Keywords:** psychedelic, history of psychiatry, psychedelic therapy, shock therapy, hallucinogen, LSD, psilocybin, mescaline

## Abstract

This article reviews the historical protocols for the administration of “classic” psychedelics in France, from the 1920s to the 1960s. Taking a chronological approach, it investigates the way mescaline, LSD, and psilocybin were administered, the subjects involved, the route of administration, the dosage, and the epistemological context of the research. From the 1930s, the Sainte-Anne school dominated French experimentation with psychedelics, inserting these studies on “hallucinogens” into a biological conception of therapeutics, where the notion of “shock” dominated. The sessions show particularly anxious experiences, sometimes described as “torture” by the patients who underwent them. With just a few rare cases of recovery reported, these substances were not considered as medicines, but rather as tools for exploration in the context of experimental research; thought of not as *psychedelics* (“mind manifesters”) but as *psychodysleptics* (“mind disruptors”). While these tools could be useful for the diagnosis of sick patients, French physicians did not manage to demonstrate clear therapeutic benefits in the use of psychedelics, perhaps because of their reluctance, in most cases, to determine an optimum dose, and also very often to appreciate the context of administration and the relationship with the patient. This article allows us to understand the reasons for the therapeutic failures reported by these early French psychedelic researchers, but also to help explain the current reluctance of French health professionals who in the face of the “psychedelic renaissance” remain strongly influenced by the very negative early representations of these substances.

## Introduction

The study of serotonergic hallucinogens, or “classic” psychedelics, by French medical researchers, began with the peyote cactus and its alkaloids, especially mescaline, in the early 20th century. In the 1920s, the pharmacologist Alexandre Rouhier captured the attention of the French and international scientific communities, first through his important monograph on the uses of peyote, which was translated several times, and later through his pharmaceutical preparations. Mescaline, on the other hand, was first studied in France by the famous neurologist and psychiatrist Henri Ey, beginning in the 1930s. From 1951 onwards, the therapeutic properties of LSD were in turn evaluated. Then, in 1958, the Swiss chemist Albert Hofmann extracted and later synthesized both psilocybin and psilocin from mushroom samples given to him by French mycologist Roger Heim, director of the Muséum national d'histoire naturelle (National Museum of Natural History) in Paris. France was also home to the beginnings of modern psychopharmacology, when in the 1950s psychiatrist Jean Delay and his team at the Hôpital Sainte-Anne in Paris gained international recognition for their pioneering use of the first neuroleptic (antipsychotic) chlorpromazine in psychiatric care.

Despite these early studies of psychedelics, French scientists were quickly overtaken by the methodological and epistemological developments proposed by therapists and researchers elsewhere. Remaining committed to their conceptual framework of “shock therapies” common during inter-war psychiatry, French researchers ignored extra-pharmacological considerations such as phenomenological subjectivity, which is nonetheless decisive to the psychedelic experience and its therapeutic effectiveness. This isolationism must be understood in a context where the precepts of psychoanalysis were not predominant in France and were more theoretical concepts than models to be applied concretely with patients within the psychiatric institution. As a result, the doctor-patient relationship had not fundamentally evolved (Guillemain, 2020). A century earlier, the French alienist Moreau de Tours had however already formulated the importance of the environment and the state of mind of subjects in psychotropic drugs' experience (Snelders et al., 2006; Hartogsohn, 2017). But for most French doctors in the first half of the 20th century, these ideas were in the same way not transposable to the case of patients. In this theoretical context, the few cures reported in this early psychedelic literature seemed more to be happy accidents rather than the fruits of an intentional and skillful administration of these substances.

We propose a historical analysis of the theoretical and practical conditions of the administration of psychedelics on humans in France. The “psychedelic renaissance” reflected in the renewed interest in the therapeutics of these substances over the last 20 years in North America and Europe, has been slow to materialize in the form of French clinical trials. We observe a historiographical issue relating to the often-mixed results of clinical and experimental psychedelic usage in French psychiatry, compared with some of the results obtained abroad. It seems essential to question the reasons for the poor therapeutic results observed by French teams in their use of psychedelics. We hope that this work enables current therapists and scientists to better understand and question their own practices and to recognize when aspects of their clinical failures are owing to commitments to certain methods used in the past.

This article is organized in three parts, examining the specific research carried out in France using mescaline, LSD, and psilocybin respectively. By identifying and analyzing the administration protocols, dosages, environmental conditions, conceptual frameworks, and other factors used in this research, we intend to describe the specific character of French psychedelic research in comparison with approaches developed elsewhere in the world.

## Mescaline

In France, in the 1920s, it was first all of peyote's active component—alkaloids—that were considered potentially helpful as medicines. Of all these alkaloids, the identification and synthesis of mescaline became most important and was experimented with in isolation in the 1930s and 1940s by great names of French psychiatry of this era: Henri Claude, Henri Ey, and Jean Delay, working out of the legendary psychiatric hospital and asylum Sainte-Anne in Paris. A look at the context in which these first psychiatric studies using mescaline took place allows us to consider the later reception of LSD and psilocybin in French medical and pharmacological circles. Indeed, French psychiatry was undergoing a neurobiological turning point in the 1930s, which these studies on mescaline illustrate and support.

### a. Alexandre Rouhier: the totality of peyote's alkaloids, for a polyvalent therapeutic action

The 1927 appearance of pharmacist Alexandre Rouhier's *Le Peyotl: la plante qui fait les yeux émerveillés* (*Peyote: The Plant That Fills the Eyes with Marvels*) was a success considering its somewhat obscure subject matter (Rouhier, 1927). He proposed a complete synthesis of existing publications on the cactus, relying on a relatively abundant but hitherto scattered anthropological literature. He also self-experimented with peyote and its different alkaloids to give a description of their precise physiological and psychological actions.

From the book's outset, Rouhier insists on peyote's therapeutic potential. Peyote buttons, and later mescaline, had been experimented with by medical researchers as early as the late 1900s. But Rouhier, following the German chemist Arthur Heffter, proposed a comparative study of the physiological effect of many of its constituent alkaloids, including mescaline but also anhalonidine, peyotline, anhalonine, and lophorine (Perrine, 2001). He thought the convey between the alkaloid content of the plant and the dosage of pharmaceutical preparations, especially of pure mescaline.

“English and American experimenters mention only the number of “buttons” or the weight of the pharmaceutical preparation employed, without relating them to any alkaloid content. Heffter's test and ours alone allow us to estimate that 0.20 grams of pure mescaline or 0.75 grams of total alkaloids, represented by their equivalent in dry drug or in extracts, are necessary to obtain the visual manifestations of sacred intoxication and to trigger the process of concomitant phenomena.” (Rouhier, 1989)

Finally, Rouhier reflected on “the necessary dose” for different uses, such as for the purposes of its therapeutic effect or what he referred to as “sacred intoxication”. He wrote that “the dose necessary to produce ‘sacred intoxication' was much higher than that considered therapeutic” and that this higher dose had “never been perfectly established”. Indeed, the concern over what constituted a reasonable dose capable of producing these visual effects emerged in 1896, when Heffter identified mescaline among peyote's alkaloids as responsible (Perrine, 2001). To determine this efficient dose, Heffter consumed mescaline very gradually and with great caution, from 20 to 150 mg, equivalent to the mescaline alkaloid content of 2–5 buttons. The anthropological literature, with which Heffter was familiar, reported a wide range of the numbers of buttons consumed, from a few to about 30 buttons (According to ethnologist James Mooney's observation among the Kiowa of Oklahoma) (Rouhier, 1989). Not long after, in the first experimental protocols of mescaline in psychiatry by Alwyn Knauer and William Maloney in New York, the use of 200 mg became normalized (Knaueur and Maloney, 1913).

Rouhier took an altogether different approach, favoring pharmacological preparations of extracts that included the totality of the peyote alkaloids: he was attentive to the variety of Indigenous therapeutic uses and envisaged numerous applications for Western medicine. The dosage was variable according to the indication. Rouhier reported personal experiments with small doses, or more significant doses, going from 40 to 1,000 mg of total alkaloids. In small doses, he noted “a very appreciable physical and mental overactivity,” while with large doses, vivid and intense visions are at the heart of the experience (Rouhier, 1989). “Quite massive doses of plants” could, he wrote, serve as a “mental detonator.” He advocated such doses in experimental psychology and psychoanalysis. He produced several preparations in the form of powder, tincture, fluid extract, and soft extract or injectable solution. The soft extract, in tablets, and the injectable solution were marketed under the name of *Panpeyotl*, starting in 1927. The tablets of *Panpeyotl* contained 250 mg of plant extract, 33% of which he believed was comprised of the alkaloids; a single tablet thus corresponded to a very weak dosage. He describes giving the subjects of his experiments 8 tablets, or 2,000 mg of extracts. One year following the publication of Rouhier's work, the physician Raymond Briau devoted a medical thesis to the role of peyote in the treatment of anxiety. He used Rouhier's *Panpeyolt* preparation but was very careful about following Rouhier's protocols in his own study:

“The dosage had to be fixed. And we should not be surprised to have used low doses, too low perhaps, in our first tests. It is that we proposed to determine the useful minimum, seeking in fact to reach, to calculate it, not the obtaining of peyote intoxication in its most complete phase including hallucinatory phenomena, but rather the realization of a pre-ebrious state corresponding to the first phase of mescaline intoxication, according to Rouhier.” (Briau, 1928)

Briau experimented on nearly 20 subjects presenting with anxious states, at the hospital and who were for the most part interned, with doses that varied from 250 mg to 1,500 mg of *Panpeyolt*. The doses could be repeated over several days, not consecutive, according to the patients' general condition. The mode of administration was generally in pill form, but some patients were given injections. Following Rouhier, Briau recognized that the drug's action was more intense by hypodermic route, but also shorter in duration. He also admitted to having used doses that were too low, out of caution, but also because he had limited quantities at his disposal. Finally, he expressed optimism about peyote's effect on anxiety, because of the harmlessness of the substance (unlike opiates) and its hypotensive (blood pressure reducing) action. Despite his tentative enthusiasm he feared that “the future of the drug would be limited”, due to “the scarcity of plants and their easy depletion if their harvesting became intensive” (Briau, 1928), and he expressed the hope that the active ingredients could be synthesized and used separately, to gain even more effectiveness.

As late as 1956, a Parisian pharmaceutical laboratory was still marketing a Rouhier-like preparation—*Peyotyl*—as a sedative recommended for overwork and depression. In addition to peyote, this drug contained belladonna and henbane extracts and phenobarbital. It was described in a promotional leaflet as: “a sedative and regulator of the vago-sympathetic system, cerebral stimulant, euphoric, antispasmodic, analgesic, antithermal PEYOTYL is a factor of balance, wellbeing and calm; it gets a sensation of relaxation while increasing the intellectual activity.” This concoction of multiple components, which could be described as polypharmacy, exhibits an ancient conception of medical remedy, as panacea, which Rouhier strongly believed. His interest in the occult and alchemy certainly explains this conception. However, this particular vision of psychopharmacology was very far from the biological psychiatric approach to medicine that emerged in the 1930s.

### b. “Hallucinogenic substance”

In 1919, German chemist Ernst Späth obtained the synthesis of mescaline in the laboratory. It became a “pure white drug” (Jay, 2019), according to the historian Mike Jay, and was made available to researchers, notably in a pioneering way by the German pharmacy company Merck, in the form of an injectable solution. Mescaline now seemed easier to use, its production was more stable, and the alkaloid content more constant. In Merck's accompanying protocols, the suggested doses were increased compared to the first era of trials using mescaline extracted from peyote. British-German psychiatrist Wilhelm Mayer-Gross claimed to have “doubled” his dosages compared to the historical protocols, thus experimenting with 400 mg of mescaline (Mayer-Gross and Stein, 1926).

It was in 1934 that the first French study using synthesized mescaline was published (Claude and Ey, 1934). It was carried out by psychiatrists Henri Claude and Henri Ey, who experimented with it between 1933 and 1937. Their first publication on the subject revealed an experiment using it on both doctors and patients. Mescaline was injected hypodermically in doses varying from 250 to 500 mg, with the authors estimating that “the effective dose is generally around 0.45 g for an average weight adult”.

The paper introduced the term “hallucinogen” into the French language. The term, which became widely accepted, is particularly revealing of the tradition of thought to which the authors attached themselves and its association with the psychosis model of the mescaline experience developed by German psychiatrists in the 1920s (Beringer, 1927). The choice of the word revealed a specific emphasis of just one of the symptoms of the mescaline experience: that of visual distortions that were widely associated with the hallucinatory phenomenon and pathology.

Their approach was intended to be non-reductive to mere biological explanations however. In France, Claude and Ey were among the first defenders of conceptions of psychiatric disorders that were psychodynamic (Claude and Rubenovitch, 1940). Of course, they considered it possible that mescaline had a biochemical action. But they also held that its effects on the subject varied according to a specific and personal “psychic terrain.” They mentioned the case of one patient who had reacted very positively to the mescaline experience. The therapeutic value of the action of mescaline thus deserved to be investigated further:

“Perhaps the most remarkable case we have observed is that of a patient committed for melancholic depression with sensations of depersonalization who recovered her personality, the normal bodily impressions during mescalinization. She was discharged cured a few days later.” (Claude and Ey, 1934)

### c. Mescaline and biological shock

Despite these early accomplishments, the study of mescaline remained limited in France in the 1930s and 1940s. At Sainte-Anne, it was not until the end of the 1940s when LSD began to be experimented with internationally that mescaline studies took off again. In those intervening years, “biological therapeutics” was approached from the point of view of “shock”. As early as 1940, Claude and Pierre Rubenovitch had established an inventory and a research program for their discipline (Claude and Rubenovitch, 1940). Therapeutic techniques used since the 1930s, insulin and electroshock therapy, were described and commented. The theoretical framework was the common idea of a shock, which was believed to put the patient into a new state, and to induce an easing or lifting of the pathological symptoms.

At Sainte-Anne, the first studies of mescaline were carried out by Dr. H. P. Gérard, in Jean Delay's department. In 1948, an article reported on more than 50 experiments conducted since 1946 (Delay and Gerard, 1948). This article is of great help in understanding the choices made for the implementation of subsequent protocols, as its method of administration and dosage were clearly outlined and explained. Delay and Gérard first set out to compare the duration of latency between administration and the manifestation of psychophysiological effects in different subjects *via* either *per os* ingestion or intravenous routes. They noticed that the effects seemed to occur more rapidly by the intravenous route, with reactions as early as 20 min after administration. The oral route resulted in a slower and rather variable reaction, manifesting from 30 min to 6 h after ingestion.

The dosages also appear to have been experimentally determined: the same subject thus received three doses of mescaline at distinct periods, first 200 mg which “gave a state of drunkenness with motor excitation, duration of mydriasis [pupil dilation] 3 h”, with 350 mg there were “slight psychosensory disorders, and the mydriasis persisted for 6 h”, finally with 600 mg the doctors noted “flushes of onirism [hallucinatory states] and the mydriasis was still sensitive 16 h after the injection” (Delay and Gerard, 1948). With clinical observations attesting to the great physiological variability between subjects, the doctors nevertheless established a fixed dose-per kilogram standard of “7 mg at the beginning, 9 mg thereafter”. The absence of constants in the results was, for the authors, quite remarkable.

Mescaline's use for the exploration of the personality remained impossible; the psychosis model prevailed. Delay and Gérard's subsequent publications placed even stronger emphasis on the phenomena of illusions, hallucinations, and the synesthesia encountered during the mescaline experience (Delay and Gerard, 1948, 1950; Delay et al., 1949, 1951). However, their efforts were not only descriptive or phenomenological, but part of a strictly biological reading of the etiology of mental illness. Illusions and hallucinations were considered the result of an “intellectual deficit”, in relation to the subject's full or normal capacities, and also seemed useful to doctors researching the literal location of disorders in the brain. Thus, for them: “most of these modifications: lesser resistance of the forms, blooming of the pareidolia, prove a regression of the perception to a lower level” (Delay and Gerard, 1950). In 1953, Diane Allaix defended her medical thesis on the psychopathology of “mescaline intoxication”, based on the experiments of Delay and Gérard (Allaix, 1953). Her “very risky” but cautious conclusion posted “the anatomical localization of mescaline intoxication phenomena” and hypothesized the main area of action to be the diencephalon.

From the experimental point of view, these mescaline studies contributed to the first hypotheses in the biology of mental illness and the location of brain disorders. After the discovery of chlorpromazine and its antagonistic effect on the action of hallucinogens in 1952, the following protocols for experimenting with mescaline became more specific: administration was reserved for patients for the purpose of studying its psychopharmacological modalities. Indeed, the Sainte-Anne team published a pioneering work on the mescaline/chlorpromazine antagonism (Delay, 1956a). In 1956 Delay and his team conducted an experiment with 37 male patients, a group composed of a majority of schizophrenics but also of patients suffering from other pathologies as manic-depressive psychoses (Delay et al., 1956). Mescaline hydrochloride was injected, slowly, at a dosage of 10 mg/kg body weight, the subject “fasting, lying down, isolated in a room with an observer [therapist].” The objectives of the mode of administration modalities, by intravenous route on the fasting subject, were clear: it was a question of inducing an important biological modification, a “shock” of the system. The constant presence of the observers was to perform “systematic measurements (which) were regularly carried out immediately before the injection, 10 min afterwards, then half an hour, 1 h, 2 h, and 4 h afterwards,” sometimes encephalographic or polygraphic examinations completed the battery of tests carried out. In addition to the biological modifications measured, Delay and his team concluded that “two phenomena” were common to this study: anxiety and difficulty of contact.

Around this time in the east of France, psychiatrist Marie-Thérèse Wilhelm was questioning the context of mescaline administration to which the patients were subjected, and which could understandably justify the reticence, “anxiety and difficulty of contact” described by Delay (Wilhem, 1955). In her doctoral thesis, she described her large-scale protocol involving 80 observations of mescaline and sick patients. She asked for their “the full consent”, which “often made it possible to establish an excellent contact and a climate of mutual trust”. Her objective was to study the effect of mescaline on different pathologies: schizophrenia, manic-depressive psychoses, delusions, chronic delirium, and dementia syndromes. Employing a similar protocol of 10 mg/kg body weight in fasting patients, she noted variable reactions according to the patients and their pathologies, some showing enthusiasm when faced with the experiment, the anxiety being less pervasive than in Sainte-Anne. Her conclusion however retained the exaggerated claim that mescaline induced “psychic disorders in the very sense of psychosis”. Wilhelm's interest in mescaline was undeniably diagnostic, perhaps even prognostic: aggravating the clinical picture, the substance could become a tool of definite help for the psychiatrist, from the nosological point of view.

### d. Therapeutics through “induced psychosis”

In France, mescaline was above all utilized in experimental studies. However, the question of its use as a therapeutic adjuvant was raised again by a team in Nice led by Dr. Paul Cossa. In a paper published in 1956, the team explained the protocol and its stakes as follows:

“The principle of the method is quite simple: artificial psychosis is induced by the drug, then abruptly stopped after an hour or two by a high dose of chlorpromazine, in the hope that the disappearance of the delusional and hallucinatory phenomena, artificially induced, will be accompanied by the disappearance of the previous psychopathic disorders that the subject might have presented.” (Postel and Coss, 1956)

The association of mescaline and chlorpromazine was influenced by American psychiatrist Herman Denber, who presented his research and its positive results in therapy at a Congress of French-speaking alienist physicians and neurologists held in Nice in 1955. The protocol set up by the French doctors was identical to those used in a study carried out in New York by Denber and Sidney Merlis (Denber and Merlis, 1954). The patients received 500 mg of mescaline sulfate intravenously, and after about an hour-and-a-half an injection of 50 mg of chlorpromazine was given, this time intramuscularly. The subjects in both studies also consisted mainly of interned patients, the majority suffering with schizophrenia. These consistencies aside, the recovery of the patients were divergent and the statistics contradictory; the French study showed “disappointing” results. Why such a difference?

First, Denber did not operate with the notion of “mescaline-as-experimental psychosis,” and postulated more an interest on mescaline as an adjuvant to psychotherapeutic analysis. However, this concept of “induced psychosis” was featured in the title of the article by Cossa's team. Moreover, Denber's protocol included a long psychotherapeutic follow-up, including to see the patients the afternoon of the day of the mescaline experience, the next day, then once or twice a week for as long as possible. This approach reflected a therapeutic relationship, and the self-experiences of the members of the team allowed empathy with the subjects. In Nice, patient follow-up was probably shorter, its duration perhaps not allowing for sufficient hindsight; many of the experiments dated from the end of 1955, and were reported in July 1956. In their conclusion, the authors themselves questioned “the operating mode” (Postel and Coss, 1956).

The modus operandi was in question, as was the conceptual framework. Indeed, Cossa was motivated by Delay's formulation of biological shock, which is quoted extensively. The actual conditions being faced by the patients, their psychological improvement, or their cure after the experiment, were only very briefly considered. Delay had suggested that the fact that patients were generally anxious during mescaline experiments represented an obstacle to the improvement of their conditions (Delay et al., 1956). But from a Freudian perspective it was precisely the anxiety caused by mescaline, and the way in which this anxiety could be supported and analyzed in a therapeutic process, that Denber valued (Denber, 1956).

Another experiment with the mescaline/chlorpromazine alliance was managed at Sainte-Anne, by a young doctor, Martine Ropert, doing her thesis under the direction of Delay in 1957. This was the last mescaline protocol at the hospital. Following the same dosages as the team in Nice, and particularly aware of Delay's previous work, as well as of international research, Ropert concluded her study in these terms:

“If we compare the therapeutic results (of whatever degree) of simple mescaline shock and the mescaline-chlorpromazine association, we see that it is above all in the ‘improvement of contact' group that the positive effects of this association are to be found, whose interest, we repeat, seems to us here to be more psychotherapeutic than biochemical.” (Ropert, 1957)

However, the therapeutic relationship was hardly investigated at Sainte-Anne. Although Ropert acknowledged the necessity of “a reassuring contact” with subjects to ensure the good progress of the mescaline experiments, her descriptions of both during and after the experiments, as well as patient follow-ups, affirm the pre-eminence of the biological framework at the expense of the patient/doctor relationship. The example of *Observation 8* is thus revealing. The patient was a 25-year-old man with a “mental automatism syndrome” and “ideas of persecution”. It is specified that in the service “he remains anxious, isolated, reticent…. contact with the doctors is dominated by embarrassment, distrust”. He received a first intravenous dose of 600 mg of mescaline on December 20, 1955. A quarter of an hour later he expressed his anxiety:

“I can't take it anymore. These are experiments that you make, I don't know if you'll succeed. I'm in pain, I'm aching all over, I feel bad. I'm afraid of going crazy, of not coming back. I'm afraid of going crazy, of dying. To become crazy, it's to see such things. They are impossible to tell... they don't look... it's not human anymore. You're afraid you won't be normal again, you're afraid you'll die. You should stop this, you're beyond your means, you're beyond human possibilities. You don't know what you're doing. It's been going on for centuries.” (Ropert, 1957)

Ropert noted that a week after this experience, the patient showed a reluctance regarding the experience, with a strong fear that it would be repeated. The patient was then subjected to a insulin shock treatment in January 1956. On March 22, and against his objections, he underwent a second intravenous injection of 500 mg of mescaline, followed an hour-and-a-half later by an intramuscular injection of 50 mg of chlorpromazine. A week later, he began a daily course of neuroleptic medication, with an oral dosage of 8 mg of reserpine (a neuroleptic). During this treatment, a third experience of mescaline was imposed on him, this time a strong dose of reserpine had been given to him before. His case shows how patients undergoing psychedelic treatments were also subjected to a whole arsenal of biological treatments.

## LSD

The study of LSD in France began in 1951. From the beginning, French research was characterized by a commitment to low doses.

### a. At the outset, some innovative proposals

In 1953, French neurologist and epilepsy specialist Henri Gastaut studied the effects of LSD on the human brain using an electroencephalogram. The study was conducted on 12 “normal” men between 25 and 50 years old, with an oral dose between 40 and 60 μg. In addition to their experiments on the brain, Gastaut proposed curative hypotheses linked to the administration of LSD. By altering mood and increasing the rate of information experienced by the depressed subject, he saw “important therapeutic consequences” to the use of the substance. He supposed that a dose “just liminal, and perhaps infraliminal (i.e., imperceptible, of the order of 0.25 gammas per kg of weight)” would improve subjects' psycho-affective and psycho-motor behavior. LSD could thus be a modifier of a person's “affective tone” at doses imperceptible to human consciousness. He also noted that “If, until now, success has not crowned all the therapeutic trials carried out with LSD 25, it is probably… because they involved excessive doses” (Gastaut et al., 1953).

Four years later in 1957, psychiatrist Daniel Widlöcher published the first thesis in France on LSD (Widlöcher, 1957). Although his female patients reported feeling more anxious than euphoric (five anxious cases compared to 4 euphoric), the young doctor believed that by varying the experimental conditions it was possible to improve patients' responses and reduce their anxiety. Widlöcher noted patients feared being left alone and insisted that “psychological preparation seems to play a role and in cases where the patients had the opportunity to be better informed and better prepared for the experiment, it went more smoothly”. He criticized the expert status of the psychiatrist in the management of the sessions: “The psychiatrist will *himself* learn from the testimonies of the subjects under LSD to possibly modify his attitude toward the patient”. By listening to the patients' descriptions of their own feelings during the experiment, “we will learn to know better the attitude to adopt toward these patients” he wrote, calling for a reconsideration of the “classic attitudes” of therapists caring for people under the influence of LSD. Widlöcher was perhaps directly targeting his thesis supervisor, psychiatrist Jean Delay, who showed little empathy for his patients (Dubus, 2023). Finally, he discussed the impact that the practice of experimental psychosis could have for the evolution of care for the mentally ill: “It frequently happens that acute psychotic patients are placed in similar conditions [solitude, darkness, inaction]. These classical attitudes probably deserve to be partially revised, if we consider what experimental psychosis teaches us.”

These considerations marked a step forward in French psychedelic therapy, however Widlöcher continued to believe that high LSD doses were unnecessary to benefit patients and noted that low doses allowed for stronger control of the situation and better verbalization by patients. Strongly influenced by psychoanalysis, in a French context that was rather opposed to this approach to psychotherapy, he particularly valued the exchange and intersubjective relationship with the patient. Widlöcher insisted on repeated sessions so that the emotional abreactive manifestations caused by reliving the past could be brought to the fore, at the expense of psycho-sensory effects alone, which tended to most impress subjects first encountering the substance. He added that only psychotherapy and joint analysis between the therapist and the patient could ensure “the happy effects of LSD's action.”

### b. Experiments at Sainte-Anne and Bonneval: toward a definitive method

The hypotheses of Widlöcher, who left Sainte-Anne immediately after his doctorate, did not appear to influence his thesis supervisor, nor Henri Ey, in their own experiments on LSD.

Delay was among the French psychiatrists most involved in research on shock treatments, which designed to break down the mental structure of patients in various ways to “reconstruct” it non-pathologically. In the 1940s he examined narco-analysis and amphetamine shock, a method that plunged subjects into a half-sleep by the administration of barbiturates or amphetamines and who were then prompted to involuntarily express their memories and thoughts. Patients did not consciously participate: therapists used questions to uncover information that was hidden in ordinary states of consciousness. Patients referred to this method as the “truth serum.” Rather understandably, these techniques posed important ethical problems, which were raised when Delay presented it to the Société médico-psychologique in 1946 (Delay and Shentoub, 1946). How far should a psychiatrist push in his exploration of the psyches of patients at his mercy? The psychiatrist Henri Baruk, who condemned these methods, described them as “a rape of the personality” (Baruk, 1950).

Placing LSD sessions in the same category as narco-analysis and amphetamine shock therapy, Delay proposed the term *oniro-analysis* as “onirique” means “related to dreams” (Delay et al., 1947). In this therapeutic model, whatever the substance the intention was the same: to provoke a shock to force the patients to surrender (Delay, 1951). Ey, who also adopted this conception of LSD use, noticed in 1959, underlining this information in the text, that in certain cases of neurosis, patients expressed “very traumatic past situations that she had never before revealed during previous hospitalizations” others “until then hidden” (Ey et al., 1959). He also avoided high doses, being satisfied with administering 1 to 2 μg/kilo and often not exceeding 100 μg per session. Whatever the psychic “shock” being produced, it was not equivalent to the mystical psychedelic experience described by American therapists. Delay's approach was to simply let the substance take effect for an hour or two, then end the session with an injection of chlorpromazine. The aim was to create a state that allowed access to patients' hidden psychic material but not to guide them through a transforming experience. For Delay and Ey, LSD made it possible to carry out an in-depth examination of the subject, but did not present any real therapeutic interest.

These researchers also preferred to use intravenous administration, whereas many therapists had throughout the 1950s been using the oral administration, which allowed for a more gradual onset of the effects, which was less brutal for the patients. It is interesting to note however, that French doctors administered LSD orally during their own self-experiments.

This commitment to the shock therapeutic model in France made it an outlier in LSD experimentation in this era, with most international researchers abandoning the framework from soon after the first LSD studies. As early as 1949, Swiss psychiatrist Gion Condrau had concluded that he had been unsuccessful in creating a real shock using it. His own studies included doses as large as 280 μg, which he would not consider moving beyond for fear of producing too strong disturbances (Condrau, 1949). The following year, American researchers Anthony Johnson and Warren Busch insisted on an essential difference between shock therapies and those using LSD: in this last model, they noted the absence of confusion of the subject and therefore his or her active and conscious participation, i.e., voluntary (Busch and Johnson, 1950). Meanwhile the Italian psychiatrist Rodolfo Belsanti wrote in 1952 that even with remarkably strong doses (up to 480 μg), shock played no perceivable role: “Concerning a possible shock-type therapeutic action of LSD, my impression is that it must be totally excluded” (Belsanti, 1952).

We are thus struck by the absence of reflection within the French teams working with LSD concerning the notion of “set and setting”, which had been emerging particularly in Anglo-American countries since the mid-1950s. Although most of them practiced self-experiments, this did not lead to a questioning of the protocols, as was the case with other therapists. French practitioners engaged in the study of LSD are distinguished by a distant, almost insensitive approach to the patients (Dubus, 2020); the “guinea pig” status of their experimental subjects is also felt in all their work. Delay was aware of the research conducted by other therapists that aimed at giving subjects a positive and transformative experience, but this did not seem to interest him, even writing about his own work that “the possibility of a rich hallucinatory experience or of unspeakable beauty is not frequent for the doses used” (Delay and Benda, 1958). Elsewhere he describes the effects experienced by his patients on LSD in a sub-section entitled “torture”.

The early psychedelic French scientific paradigm was thus particularly unique and tinted with nationalism compared to the international research of the time, in effect ignoring the appearance of new theories (in the field of psychiatry but also more broadly at the philosophical level) on psychedelics. This indifference to psychedelic thought outside of France is memorably captured in an article by Delay in 1956 that described Aldous Huxley as a “humorist”, and his just-published essay *The Doors of Perception* as “science fiction” (Delay, 1956b). A more specific study remains to be carried out to investigate this isolationism or even this hostility of at least a part of the French medical profession to theories developed elsewhere.

### c. Delay's influence

French doctors were thus confronted with the insoluble paradox of maintaining a paradigm that understood LSD as a *psychotomimetic*—psychosis mimicking, which at its worse meant violent and pathological—and yet still cling to the possibility that these phenomena were therapeutic. However, during the 1960s, other teams tried to evaluate the therapeutic value of LSD to treat two types of patients: men with alcohol-resistant addiction, one of LSD's main indications at the time, and homosexuals, who were then considered as mentally ill who needed to be cured.

In 1960, Dr. Roland Lanter, in his psychiatric hospital in Rouffach, in northeast France, began his experiments with alcoholics (Lanter et al., 1962). He administered mescaline or LSD in high doses via intramuscular injections ranging from 100 to 400 μg for LSD, and 600 to 1,400 mg for mescaline. Lanter considered that below the minimum indicated doses, “there are often only simple neurovegetative reactions”. According to the case studies presented, many patients took refuge in mutism as a defense mechanism. In Anglo-American countries, LSD doses in cases of alcoholism were generally 200 to 600 μg (Cohen, 1966; Dyck, 2006). Some authors mentioned doses of 1,000, 1,500 and up to 2,000 μg but these high doses were rare, and many therapists questioned the appropriateness of these quantities, as can be seen in particular in the discussion following the paper by Edward Baker in a conference in 1965 (Baker, 1967). In Lanter's unit, therapists practiced the “disgust cure”: traditionally, the aim was to induce disgust in alcoholics by associating the intake of alcohol with an unpleasant feeling, originally by administering an emetic (vomit inducing) drug with the drink. Lanter imagined associating the intake of alcohol with an intense hallucinogenic experience, understood as unpleasant.

In 1965, one of his interns, Jean Weil, published a thesis about treating 69 male patients for alcoholism with only LSD (Weil, 1965). A key phrase he used to describe his method was “cure by anguish”. It was a question of making the patient acknowledge and understand the “anguish” at the origin of their dependence on alcohol. To do this, it was necessary to find the “motivations of the alcoholic. It is with the aim of facilitating this research that we thought of using *psychodysleptics* as a means of revealing the more fundamental structures, of making them burst”. “Psychodysleptic”, meaning “mind disruptor”, is the terme coinned by Delay to classified mescaline, LSD and psilocybin (Dubus, 2021). These new kinds of disgust cures were described by Weil as “very traumatic”.

He explained that the panel of patients was originally larger but that after one or more sessions many of them “refused to continue a treatment that they considered too painful”. Weil's approach was to increase the doses even more than his supervisor, going up to 830 μg. He concluded that the higher the doses, the better the results (37.7% of “very good results” after 2 years of follow-up). The outcome was somewhat worse for group administration, as therapeutic contact could be made less easily than individually, with patients adopting what was interpreted by the author as “collective defensive attitudes in the form of contagion reactions, manifested by unmotivated and contagious laughter or by unanimous refusal to continue the treatment”. In a group setting, the patients thus found support to endure this treatment through anguish and to fight against these sessions. Although Weil declared that he wanted to carry out psychotherapy with his patients, he also admitted that he did not speak the same language as these men who spoke in a dialect closer to German than to French. It was therefore difficult to communicate them the way Widlöcher and other had recommended.

In his 1960 study, Lanter not only administered LSD to his alcoholic patients: two teenagers, placed in his psychiatric hospital because of homosexual behavior, were also subjected to it. The psychiatrist hoped that LSD could provoke in Michel, 15 years old, and Bernard, 18, “delirious flashes” to bring to light the “fundamental fantasy structuring the morbid personality”, and to push them toward heterosexuality. Lanter used the same protocol as he had with alcoholics: the psychedelics were injected intramuscularly or intravenously, in high doses ranging from 200 to 1,400 mg for mescaline and 100–800 μg for LSD. In 1959 Sutter and Pélicier recommended not to exceed 500 mg when administering mescaline (Sutter and Pélicier, 1957); the maximum dose used by Lanter was thus almost tripled. Moreover, all the French authors recommended doses lower than 100 μg of LSD. Shock was sought: the doctors noted a “stupor” and agitation of the adolescents, their screams, the fact that they tore their sheets or clung to the people present, asking for help. Lanter does not describe trying to reassure his patients, instead recommending that doctors “adopt an ‘aseptic' attitude” to facilitate the “demystification” of the behavior of these “sexual perverts” (Dubus, 2022).

### d. Another minority path

If the orientation chosen by the Sainte-Anne school, represented by its two great personalities, Henri Ey and Jean Delay, had profoundly marked the method of using psychedelics in French psychiatry, locking it into the models of experimental psychosis and therapeutic shock, some French therapists were nevertheless sensitive to the techniques of set and setting.

In 1961, the psychologist André Virel conducted his first “mental imagery” experiments under LSD with an academic group named *Groupe d'études du Rêve Éveillé Dirigé* (Directed Daydreaming Study Group) (Desoille, 1963). Ten years later, Virel published an article describing their protocol more precisely: while patients might receive doses of 100 μg by injection, the scientists only took small doses of 10 to 30 μg orally during self-experimentation, before experimenting with larger doses, up to 300 μg (Virel, 1971). Later, Virel carried out experiments with three subjects submitted to two LSD sessions starting with a dose of 25 μg with more to follow “calculated by the subject's response to the low dose”. According to Virel, the *Groupe d'études du Rêve Éveillé Dirigé* did not achieve good results and “had to fail for wanting to direct subjects' mental imagery too much”. Virel would later use music (classical and African) during sessions with his subjects and was satisfied with the therapeutic results.

Virel differed from Delay by his care to avoid producing a shock: “By proceeding by progressive doses starting from the smallest, one arrives in two or three sessions to determine the optimal dose for a given subject without having at any time risked triggering the ‘shock' signaled by the use of medium doses”. The number of patients treated in this way is not known since Virel also took care of patients outside his office. A psychologist associated with Virel but who did not participate in the group's LSD sessions, thus remembers Virel taking his patients to his house in Normandy for a weekend to administer the substance in a “more pleasant” natural setting (Odile Dorkel, personal interview, 02 November 2022). The sessions there were supervised by a doctor named Jean-Claude Benoît, who had been Delay's intern. Benoît's publications on LSD indicated his desire to work on the precepts of set and setting, but the hospital setting at Sainte-Anne did not allow him to implement them (Stévenin and Benoit, 1960; Benoit, 1963). These unorthodox practices held at Virel's house were not published.

Another French therapist seems to have been truly influenced by the precepts of set and setting and to have distinguished himself from Jean Delay's therapeutic model. This is the psychiatrist referred to by his colleague psychiatrist Jean Thuillier in 1981 as “Bernard P.” Despite numerous searches, we have not yet been able to discover any archives concerning this key figure in the history of LSD in France. We therefore only know about his practice through Thuillier's testimony: we do not know where he worked, what doses were administered, for what type of patients.

If some French psychiatrists were inspired by psycholytic theories, for example by administering progressive doses, only Bernard P. adopted the psychedelic therapy as Thuillier tells us:

“It is this last technique that my friend Bernard P. used with good results. I often attended his experiments and helped him to control them. He obtained remarkable cures in certain cases of neurosis and psychosis, by using the phase during which the subject subjected to the L.S.D. was in a state of hyper-suggestibility. The patient, who was then reliving the dramatic event that generated his illness, could free himself from it during an emotional discharge, and even more easily since he was at this precise moment deprived of any critical spirit and accepted everything that his doctor suggested to him as the primary truth.

This was really the best part of L.S.D., this remodeling of a consciousness, first destroyed or emptied, washed, then reconstructed and furnished with the good word of the psychotherapist that the patient never thought of questioning.” (Thuillier, 1981)

One senses in Thuillier's account the influence of shock therapy still present in the conception of the treatment (notably *via* the idea of “reconstruction”); this inability to think of the effects of LSD outside this framework caused a serious misunderstanding between the two friends, leading in part to Bernard P.'s suicide, an important event in the medical history of LSD in France. From then on, not only did the most influential French therapists fail to find therapeutic value in the substance, but it was blamed for disturbing some individuals so much that it drove them to suicide.

## Psilocybin

It was in France that the first medical experiments with the mushroom alkaloid psilocybin took place. Anne-Marie Quétin's medical thesis of 1960, describes how eminent mycologist Roger Heim, director of the *Muséum national d'Histoire naturelle* (MNHN), personally arranged for psilocybin to be sent to Sainte-Anne, both in tablet form and as an injectable liquid. It was sent to Delay's department by the Sandoz laboratories in Basel, beginning in July 1958 (Quétin, 1960), which is to say very quickly after Albert Hofmann and his colleagues had identified, extracted, and synthesized the Mexican sacred mushrooms' active principles psilocybin and psilocin, publishing their results in 1958. Psilocybin itself has an earlier entry into France, however, beginning with the ingestion of cultivated mushrooms for scientific research purposes.

### a. The preliminary self-experiments and the concern of the dose

The first documented intentional ingestion of psilocybin mushrooms outside America were in France by Heim himself. In the mid-1950s he had accompanied Gordon and Valentina Wasson's expeditions to southern Mexico in search of psychotropic mushrooms (Heim, 1956). Heim had previously been interested in the syndromes of intoxication with mushrooms, eating samples of fly agaric in 1924 and experiencing “hallucinations in black” (Heim, 1924, 1978). His self-experiment with psilocybin mushrooms happened on May 18, 1956, shortly after he received a letter from Mexico from ethnomycologist R. Gordon Wasson. Wasson wrote to him that he had finally experienced the famous sacred mushrooms of Mexico—*Teonanácatl*. Wasson warned: “the effects of these mushrooms are beyond belief!” (Wasson, 1955). Heim had received samples and spores from Wasson a few years earlier, managing to cultivate them in the MNHN's cryptogamy laboratory. On the fateful day, he ate 120 grams (five specimens) of fresh *Stropharia cubensis* at his home. Presumably, the dose was chosen arbitrarily: it does not appear to correspond to an Amerindian use, since the *curanderos* and *curanderas* of Mexico only consumed them in pairs, and especially since this particular species, probably imported to the American continent by the European colonizers, was the least valued (Wasson and Wasson, 1957). Whatever their relative quality, Heim later described having consumed “twice too many specimens” (Thévenard, 1964), during that first experience, which led him to “cling to the fireplace” (Heim, 1957).

The quantity of mushrooms ingested in self-tests and experiments on healthy volunteers were adjusted downwards to align with those used by Mexican practitioners, following Heim's trip to Mexico in July 1956. Thus, for an experiment with *Psilocybe mexicana* carried out in Paris on April 14, 1957, which he described as “quite notable,” he ingested 32 mushrooms, knowing that Wasson had learned that Mazatec *curanderos* could consume up to 15 to 20 pairs (Wasson and Wasson, 1957). Heim considered 16 pairs to be in the low end of the doses reserved for *curanderos*. In a passage devoted to this species in the French documentary film *Les champignons hallucinogènes du Mexique*, filmed in 1961, he states that “it takes 35 specimens to experience the optimum hallucinatory effects” (Thévenard, 1964).

It is understandable that after his first experience with a relatively high dose, Heim came to question the exact, or optimum dose. He was also attentive to the diversity of responses across individuals after consuming *Psilocybe caerulescens* during a collective ceremony in Mexico with other Westerners, after which he described an “exceptional sensation of wellbeing”, a “remarkable lucidity”, an aptitude for “a rare cerebral and physical activity” (Heim, 1957).

Heim's assistant at the museum's cryptogamy laboratory, the mycologist Roger Cailleux, whose expertise in the cultivation of hallucinogenic Mexican mushrooms proved essential, also carried out experiments, but using very small quantities (Heim et al., 1958). Perhaps impressed by Heim's accounts of higher doses, he thought it would be interesting to try and approach *P. mexicana*'s threshold of inactivity. He began by absorbing just three dry medium-sized mushrooms, weighing 0.25 g for his first experiment, 0.5 g for his second. These tests with small doses allowed him to conclude that the action of this species can be translated into purely visual phenomena, without manifestation of “any index of depersonalization”: it was indeed a question of exploring the phenomenology of the experience according to the dose. He carried out a third test with 2 grams of dry *P. semperviva* and regretted not having felt a “happy clarity of mind and exceptional wellbeing”, but rather “an indefinable malaise, similar to that which can follow a nightmare”.

Together with the ethnographic data collected in Mexico and the reports of preliminary self-observations made in Switzerland by Albert Hofmann et al. at Sandoz with Mexican mushrooms or pure psilocybin (Heim et al., 1958), these Parisian reports constitute the very first information on the psycho-physiological effects of psychedelic mushrooms and psilocybin. Their experiments testify to the importance of dosage, but neither the question of the psychological preparation of the experimenter nor that of the context of the experiment appear. These data, although perceived as being of great interest for orientation, were judged “very insufficient” by Delay et al. (1958). On the one hand, because the self-experiments were judged incomplete without the careful supervision of a “psychopathological observer”—it was not a question of having a person stand-by to reassure the self-experimenter, but rather to allow the collection of scientific, objective, quality data. On the other hand, it was necessary to ensure these isolated data were gathered according to the systematization of the experiment under well-controlled conditions on enough subjects.

### b. Cautious self-experiments of psychiatrists with psilocybin

In July 1958, soon after psilocybin was produced by the Sandoz laboratories, Sainte-Anne had tablets and injectable solutions of this new substance. Jean Delay's team was then able to conduct a large study of its use, exploring its physiological and psychic action and evaluating its therapeutic action; this was, indeed, a pioneering study at the world scale.

It was designed to use the drug on both “normal” healthy volunteers and on “mentally ill” people. The very first published data on the psycho-physiological and clinical study of psilocybin, from Sainte-Anne in 1958, notes that out of 16 protocols carried out on 13 normal people, the minimum dose was 5 mg and was administered to only one subject (Heim et al., 1958). Looking closely at the protocols, there is no indication that the doses used were calculated to approximate the doses of mushrooms used by the Mexican peoples, or according to the self-experiences of Westerners like Wasson or Heim.

Most (11) received a dose of 10 mg in a single session. Among these “normal subject” trials, there were two protocols with a dosage of 11 mg, one with 12 mg, and one with 14 mg. It is very likely that most of these normal subjects were doctors themselves: indeed, we learn in a 1961 publication on the therapeutic implications of psilocybin by the same team (Delay et al., 1961), that out of 47 volunteers qualified as normal who took part in the self-test at that time, 35 were doctors. The dose administered was 10 mg, a dose that seems perfectly arbitrary, for a total of 52 protocols. Most of the volunteers tried only once, but in six cases there was a second attempt. In fact, the article states that “normal subjects were given a dose of 10 mg orally; in a few cases where the reaction was minimal, a second trial was made with a higher dose, never higher than 14 mg”. The minimum dose of 5 mg and the common dose of 10 mg were thus apparently decided randomly, in relation to the decimal numeral system, rather than to empirical data derived from the previous trials with mushrooms.

As for the notion of an upper limit not to be exceeded, it was mentioned by Delay as early as 1958 in a presentation he gave at the Congress of Psychopharmacology held in Rome. Presenting for the first time his team's psilocybin clinical trials, he explained that “most of the trials on healthy volunteers were done with 9 or 10 mg of psilocybin, only one with 14 mg, only one with 15 mg…. [I]n spite of the transient character of these states, their nature must incite us to consider this dose of 15 mg as an upper limit not to be exceeded” (Delay et al., 1959). This 15 mg dose limit of psilocybin is certainly much lower than the comparative doses used in Mexico in the ceremonies in which Wasson and Heim participated, as well as to large doses corresponding to Heim's self-experiments mentioned above. Delay and his colleagues were therefore surprisingly cautious with psilocybin.

In a 1958 letter from Dr. P. J. Nicolas-Charles to Heim, the doctor informs the mycologist that, “concerning the clinical study of the psilocybin preparation that [he] was given,” he still “does not find any hallucinosic [sic] effect” (Nicolas-Charles, 1958), so much so that the Sandoz laboratories in Basel offered to analyze the product he had used and to send him back some “fresh product”. He told Heim that Hofmann's team even doubted that “this compound [psilocybin] sums up the properties of the mushroom”. To make direct comparisons with psilocybin, Nicolas-Charles asked Heim for some mushrooms to study for himself. Although we have no information on the outcome of this attempt to compare the effects of psilocybin with those of mushrooms, the letter bears handwritten indications in pencil, in Roger Heim's handwriting: “10 g”, “0.6”, and “0.1”. Did he try to calculate the amount of psilocybin in a given weight of hallucinogenic mushrooms cultivated at the MNHN? It is surprising that in the first French medical publications on the clinical study of psilocybin, there is no trace of any reflection on the equivalence between the doses of mushrooms used in the preliminary self-experiments and the doses of pharmaceutical psilocybin used in the hospital experiments. There is certainly a strong disciplinary compartmentalization between the actors of purely medical research and the mycologists and experimenters.

### c. The influence of the context on the experience

One of the “normal” volunteers was not a doctor: the influential poet Henri Michaux was also a subject of experiment at Sainte-Anne. In an essay published in 1960 (Michaux, 1960), Michaux evokes “the extreme indecency of being under the effect of a drug in front of strangers who have not taken it”. During the experiment, he heard for example a doctor whisper into the ear of another: “typical case of depersonalization”. In a letter to Heim thanking him for allowing him to participate in the psilocybin experiment, he described the awkward presence of “four medical witnesses in a director's office…. In spite of their discretion, they created a situation which was very unfavorable to me” (Michaux, 1955). Michaux characterizes this rather disordered and intrusive context without ambiguity as “very unfavorable”, thus illustrating the French experimental approach that appeared ignorant of the role of context in allowing a positive experience. Heim and Michaux agreed that the latter could repeat the experiment at home, with a lower dose of 4 mg.

This brings us back again to the question of how to consider the extra-pharmacological parameters affecting a person's experience with psilocybin, in this case, the reassuring presence of a person—or lack thereof—who could help prevent the experience from being unpleasant. In the entry “self-observation #28” in Anne-Marie Quétin's thesis, the psychologist who entered the room to make take tests was considered “a torturer” (Quétin, 1960). Quétin stresses “*the role played by the presence or absence of a third party*,” noting that “the attitude of the subject toward the observer can be very variable, sometimes opposite”. One subject stated that “the presence of a third party is often deeply disturbing” and another commented: “I am a little annoyed by the prosaic nature of the examiner, his worries as a drudge who.... stops my momentum with questions”.

Quétin concluded: “The attitude of the doctor, his questions, the renewed physical examination... are all factors which bring the subject back to ‘reality,' momentarily suppressing the delusional belief. Therefore, we tried to reduce as much as possible these different examinations and why we tried to keep a perfectly neutral attitude during the whole test”. This conclusion illustrates the way in which the phenomenology of the experience is perceived: it is at this time a “delusional belief”, i.e., a manifestation of the register of the pathological, rather than as being psychological states that give rise to perspectives that can escape norms around what is normal and pathological. It also reveals an explicit position on the importance of the context in the psychic effects. But this “perfectly neutral” attitude, thought to allow the objectification of the effects, could be experienced by participants as painful, as for example in the case of a Spanish doctor who in 1958 underwent two self-experiments with LSD and psilocybin at Sainte-Anne and who afterwards wrote of his examiners: “They had forgotten their human nature and did not even remember the defenseless condition of the man they had in front of them” (Toscano Aguilar, 1959). The objectification of the drugs' effects thus led to the objectification of the people who took them, certainly made even more painful while experienced in the psychedelic state.

Quétin also emphasized the difficulty of subjecting volunteers to the study of biological modifications caused by psilocybin. She observed that data gathering such as multiple blood tests and the use of electroencephalography modified “considerably the test of Psilocybin” (Quétin, 1960). Some volunteers also complained about the “boredom” or even the “irritation” caused by the psychological tests that were conducted during the sessions, which required moving around the hospital, including taking stairs. To the extent that the importance of context was noted, the parameters changed for the psilocybin experiments were to avoid repeated blood tests which “‘stop' the emotional phase so interesting for the psychopathological study” and by sparing most patients from electroencephalographic testing. These changes illustrate the trial-and-error method used by the Sainte-Anne psychiatrists, who would apply corrections to the protocols as they went along.

### d. Experiments with psilocybin on the mentally ill at the Hôpital Sainte-Anne

In addition to her account of experiments on “normal subjects”, Quétin also describes protocols initially carried out on 72 patients, with a very lopsided gender balance: 64 women and 8 men, and a total of 80 protocols. Her final clinical study included 61 patients, excluding 11 because of “poor examination conditions”. The details of these failures are not given, but we know, for example, that an anorexic and depressed woman was left alone in her room after having received an injection of psilocybin. She was treated with chlorpromazine and therefore the doctors thought that her reaction would be limited to somatic modifications.

This particular case happens to constitute one of the rare instances where the patient was said to have undergone an “indisputable improvement” following the administration of psilocybin. After injecting her intramuscularly with 8 mg of the substance, they sent her back alone to her room where she felt herself “to be in paradise” and experienced a certain transcendence of time. In fact, this patient gave a written account of her experience in numerous poems with titles evoking euphoria, even mystical ecstasy: “Alleluia, Plenitude, Spring, Eden, Euphoria” (Verroust et al., 2021). The doctors stopped her treatment with chlorpromazine and 3 days later injected her again with 8 mg of psilocybin, this time under medical supervision. This second ordeal led to an influx of forgotten memories which, according to the doctors, allowed her to expose the psychogenesis of her illness and her emotional traumas. The improvement of the patient's general condition, including a regaining of weight and a “reversal of mood with euphoria” resulted in the publication of a case study.

The patients who received psilocybin at Sainte-Anne were interned and presented a diversity of mental affections classified in five groups: “schizophrenics”, “chronic deliriums”, “manic-depressive psychoses”, “mental debilitates”, “neuroses/psycho-neuroses”, and “psychic imbalance”.

The faster action of psilocybin injections led doctors to prefer it to the oral route (*per os*). The average doses used were: 9.26 mg *per os*, 8.36 mg subcutaneously, 9.07 mg intramuscularly. The doctors administered psilocybin before any treatment, except when the treatment was already underway, in which case it was interrupted, with a few exceptions such as the anorexic patient, which made it possible to study some drug interactions. Consequently, their reactions were very diverse, but the improvement to their symptoms was only observed during the duration of the experiments, or only briefly in the following days.

If the psilocybin was “presented to them as a means of release, or as an additional test necessary for diagnosis”, the fact that psilocybin could be administered on the first day of admission to the hospital suggests that the doctors did not consider it necessary to prepare the patients psychologically, beyond simply providing this information. Moreover, the reading of the 19 observations recounted in Quétin's thesis that give the most characteristic descriptions of the action of psilocybin, shows that the anxiety episodes largely dominated over the euphoria episodes. Above all, it shows that the effect of psilocybin was perceived as a exacerbating factor in the symptomatology. Thus, Quétin acknowledges the “obvious diagnostic value” of psilocybin. Its therapeutic action was admitted, given the improvements that were momentarily observed, but Quétin underlines that “the trauma that constitutes this experimental psychosis, the partial amnesia that follows the confuso-oniric phases, seems to bring a considerable handicap to the use of psilocybin”.

In their 1961 article on the therapeutic implications of psilocybin, Delay, Pichot, and Lempérière also conclude that “psilocybin can be used as a diagnostic method”. While its therapeutic interest is less developed, they distinguish however between a direct biological action on mood as well as a psychological action. Anyway, the French medical literature on the therapeutic interest of psilocybin seemed to have already come to an end and there were no further published experiments with psilocybin for therapeutic purposes. As Delay and his colleagues and students were more versed in biological understandings of mental illness, they held that the gap between normal and pathological psyches should be corrected not by psychotherapy but rather by psychopharmacology. We then come back to the question of the effective dose.

### e. “The stronger the dose”... and the better the context

The psilocybin doses established at Sainte-Anne differed from those used by an emerging figure in psychedelic research on the other side of the Atlantic, the psychologist Timothy Leary. On June 30, 1961, while still at Harvard, he wrote to Delay. In his letter, Leary strongly emphasized the therapeutic value of psilocybin. He outlined the work he had done with prisoners. His good results, and the insightful potentialities of the substance, owed according to Leary, as much to the favorable context of the experiments (supportive environment), as to the use of strong doses:

“We have, incidentally, used stronger doses than have been reported in the literature. Twenty milligrams is our standard dose and we have used up to 40 mg. The stronger the dose the more pleasant and insightful the experience. The two percent who found the experience unpleasant ingested lower doses.” (Leary, 1961)

We are not aware of whether or not Delay responded to Leary on this very significant difference between the doses used by the two researcher groups.

Another protocol carried out at Sainte-Anne over the same period also indicated a reflection on the context of psilocybin administration. It was conducted by René Robert, as part of his medical thesis (Robert, 1962). He took 27 amateur or professional artists as subjects, and concentrated on the analysis of the 183 artistic productions made during the protocol. The psilocybin was then administered to the volunteers, in tablets, according to the standard dose of 10 mg defined by Delay. At first, Robert worked within the hospital: after they ingested the psilocybin, he asked the participants to start a creative activity, such as drawing or painting. However, he soon noticed their difficulty maintaining such activities. He discusses the need to reorient the framework of his research, and he suggested carrying out these protocols outside of the hospital, at the artists' homes or studios, reassuring places considered to be more conducive to making art.

Psilocybin research in France at the end of the 1950s and beginning of the 1960s reflects a moment in the history of psychiatry when the biological causes of mental illness were being sought: there was an overlap between exploratory psychedelic experiments and the practice of medicine, which led to an objectification of the patient (Missa, 2006). In this perspective, the conditions for the emergence of more precise knowledge on the importance of context for the success of psychedelic therapies were probably not met.

## Conclusion

The Sainte-Anne school was characterized by its understanding of psychedelics through the biological approach, which in the 1950s was still dominated by the notion of shock. It is interesting to note that this limitation was not specific to only this class of substances. The historian Benoit Majerus has shown, for example, that although at the forefront of research on chlorpromazine, Jean Delay's teams were initially cautious about its administration and even “lagged behind” in comparison with other European institutions (Majerus, 2019). Thus, years after the introduction of neuroleptics, the practice of electroshock, lobotomy and insulin comas was still very frequent at Sainte-Anne as well as at Henri Ey's hospital, and were ultimately preferred to any other technique, which would have challenged the teams' habits. The inertia of daily practices within the hospital is therefore certainly at play in the difficulties faced by French therapists to modify their understandings and methods.

During the 1960s, while the theoretical effort at Sainte-Anne was increasingly directed toward neuropharmacology and biochemical hypotheses of mental illness, this paradigm shift did not result in any evolution concerning the therapeutic properties of psychedelics. The research on LSD in particular was in fact mainly aimed at underlining the value of neuroleptics. The hospital in which the greatest number of experiments were carried out with LSD in France, in Dr. Borenstein's laboratory, were only aimed at producing an effect considered as psychotic, and then administering different neuroleptics in order to study their rapidity of action, their effectiveness, etc. (Borenstein et al., 1965). Chlorpromazine was indeed considered the “glory” (Laurentin et al., 2019) of Delay and his team at the international level. Research on neuroleptics and psychedelics, carried out by the same teams, struggled to determine generalizable protocols, despite a strong methodological effort. Questions of nosology and dosage were at the heart of the concerns, but there was no consensus (Olié, 1992). In a context where evidence-based medicine was not yet established, scientific conclusions were based more on the individual expertise of a few major figures. In this biological approach influenced by Delay's precepts, French doctors did not manage to highlight clear therapeutic benefits in the use of psychedelics, perhaps because of their reluctance, in most cases, to determine an optimum dose, and also very often to think about the context of administration and the relationship with the patient (Henckes, 2008).

As far as the difficulty of integrating a new and more horizontal relationship with the patient is concerned, we can question the training of young French psychiatrists in the post-World War II period, whose specialization was poorly recognized, and whose training “was essentially marked by an ‘organicist', ‘somatic', ‘biological' conception” (Guyotat, 1987). However, in this analysis, we should not leave aside the debates on psychotherapeutic models that agitated the French psychiatric field. From the 1940s to the 1970s, a whole young generation of psychiatrists was committed to an ethical and just relationship with the patient, and to a reform of the institution, harshly criticizing biological methods and narco-analysis. Psychedelics, however, did not appear to interest this generation, constituting a relatively quiet and discrete preserve of experimental research. But it is notable that elsewhere in the world, in the context of psychedelic therapies, the implementation of “set and setting” in the 1960s was also due to a young generation of therapists, for whom the achievements of psychoanalysis, behaviorist psychology, or systemic psychology were clearer.

Of course, the position of the caregivers is at stake here, but the broader context of the French psychiatric institution at this crucial time in the 1950s and 1960s, when care was turning more toward outpatient treatment on the one hand, and when the pharmaceutical stakes of marketing neuroleptics were considerable, also explains why the experiments were discontinued. All the more so as the cultural image of psychedelics in the mid-1960s was becoming problematic. Finally, this often-mediocre historical results of clinical trials with mescaline, psilocybin and LSD conducted in France created a persistent negative representation, which still has a lasting impact on French medicine in its relationship with psychedelic-assisted therapy; to date, psychedelic studies have not resumed in the country. However, certain French medical research teams seem to be in the process of abandoning these older frameworks, since the prospect of clinical trials using psychedelics are being studied more and more and should soon be launched, perhaps with a stronger focus on the concepts of set and setting.

## Data availability statement

The original contributions presented in the study are included in the article/supplementary material, further inquiries can be directed to the corresponding author.

## Author contributions

ZD coordinated the entire manuscript. All the authors carried out the research necessary for the constitution of the corpus of sources, for their study, and for the writing of the article as well as for its rereading. All authors contributed to the article and approved the submitted version.

## References

[B1] Allaix D. (1953). Remarques psychopathologiques de l'intoxication expérimentale. Paris: Thèse de médecine, Paris.

[B2] Baker E. (1967). “LSD Psychotherapy; LSD Psycho-Exploration: Three Reports,” in The use of LSD in psychotherapy and alcoholism, Abramson, H. A. (New York: The Bobbs-Merrill Company) 191–207.

[B3] Baruk H. (1950). L'alerte. Esprit 18, 343–346. 10.1525/curh.1950.18.106.346

[B4] Belsanti R. (1952). Modificazioni neuro-psico-biochimiche indotte dalla dietilamide dell'acido lisergico in schizofrenici e frenastenici. Acta Neurologica. 7, 340–349.

[B5] Benoit J.-C. (1963). Les substances hallucinogènes. Leur rôle dans la recherche et dans la thérapeutique psychiatrique actuelles. Semaine des hôpitaux de Paris. 39, 2184–2190. 14134268

[B6] Beringer K. (1927). Der Meskalinrausch. Berlin: Springer. 10.1007/978-3-662-11451-3

[B7] Borenstein P. Cujo P. Chiva M. (1965). À propos de la classification des substances psychotropes selon leurs effets sur l'électroencéphalogramme. Annales Médico-Psychologiques. II, 429–452. 5858535

[B8] Briau R. (1928). Du Peyotl dans les états anxieux. Paris: Thèse de médecine, Paris.

[B9] Busch A. K. Johnson W. C. (1950). L.S.D. 25 as an aid in psychotherapy; preliminary report of a new drug. Dis. Nerv. Syst. 11, 241–243. 14793387

[B10] Claude H. Ey H. (1934). La mescaline substance hallucinogène. Comptes rendus des séances la société de biologie et de ses filiales. 1, 838–841.

[B11] Claude H. Rubenovitch P. (1940). Thérapeutiques biologiques des affections mentales. Paris: Masson.

[B12] Cohen S. (1966). LSD 25. Paris: Gallimard.

[B13] Condrau G. (1949). Klinische Erfahrungen an Geisteskranken Mit Lysergsäure–Diäthylamid. Acta Psychiatrica Scandinavica 24, 9–32. 10.1111/j.1600-0447.1949.tb04588.x

[B14] Delay J. (1951). “Les aveux artificiels,” in Revue des Deux Mondes., 14 n°51, p. 463–480.

[B15] Delay J. (1956a). Antagonisme de la mescaline et de la chlorpromazine*,”* in Comptes rendus des séances la société de biologie., CL, p. 512. 13343729

[B16] Delay J. (1956b). “Des médicaments psychologiques,” in Revue des Deux Mondes. p. 600–617.

[B17] Delay J. Benda P. (1958). L'expérience lysergique. LSD-25. À propos de 75 observations cliniques. Encéphale. 169–209, 309–344. 13597831

[B18] Delay J. Deniker P. Raclot M. Ropert M. (1956). La mescaline chez les malades mentaux; constatations cliniques. Ann Med Psychol (Paris). 114, 300–305. 13340394

[B19] Delay J. Gerard H. P. (1948). L'intoxication mescalinique expérimentale. Encéphale. 37, 196–235. 17941270

[B20] Delay J. Gerard H. P. (1950). Les illusions de la Mescaline. Encéphale. 39, 55–63. 15411893

[B21] Delay J. Gerard H. P. Allaix D. (1949). Illusions et hallucinations de la mescaline. Presse. Med. (1893). 57, 1210–11. 15403413

[B22] Delay J. Gerard H. P. Racamier P.-C. (1951). Les synesthésies dans l'intoxication mescalinique. Encéphale. 40, 1–10. 14849559

[B23] Delay J. Pichot P. Genest R. (1947). Le choc amphétaminique. Annales Médico-Psychologiques. 2, 272–273. 14857505

[B24] Delay J. Pichot P. Lemperière T. (1961). La psilocybine - Ses implications thérapeutiques. Sud Médical et Chirurgical. 97, 9217–9224.

[B25] Delay J. Pichot P. Lempérière T. Nicolas-Charles P. J. Quétin A.-M. (1958). “Étude psycho-physiologique et clinique de la psilocybine,” in Les champignons hallucinogènes du Mexique : études ethnologiques, taxinomiques, biologiques, physiologiques et chimiques Archives du Muséum national d'histoire naturelle, Heim, R., Wasson, R. G., and Hofmann, A. (Paris: Muséum National d'Histoire Naturelle) p. 287–309.

[B26] Delay J. Pichot P. Nicolas-Charles P. (1959). “Premiers essais de la psilocybine en psychiatrie,” in Neuro-Psychopharmacology. Proceedings of the First International Congress of Neuro-Psychopharmacology, Rome, September 1958, Bradley, P. B., Deniker, P., and Radouco-Thomas (New-York: Elsevier) p. 528–531.

[B27] Delay J. Shentoub S. (1946). La narco-analyse psychosomatique en psychiatrie. Paris: Communication de la Société médico-psychologique.

[B28] Denber H. (1956). Studies on mescaline. VII. The role of anxiety in the mescaline-induced state and its influence on the therapeutic result. J. Nerv. Ment. 124, 74–77. 10.1097/00005053-195607000-00011 13416923

[B29] Denber H. Merlis S. (1954). A note on some therapeutic implications of the mescaline-induced state. Psych Quar. 28, 635–640. 10.1007/BF01567079 13215649

[B30] Desoille R. (1963). “Rêve éveillé dirigé et LSD 25,” in Bulletin de la Société de recherches psychothérapiques de langue française, n°3, p. 46–52.

[B31] Dubus Z. (2020). Marginalisation, stigmatisation et abandon du LSD en médecine. Histoire, médecine et santé. n°15 87–105. 10.4000/hms.2168

[B32] Dubus Z. (2021). Le LSD, psychédélique ≫ ou ≪ psychodysleptique? L'Information Psychiatrique. 97, 803–808. 10.1684/ipe.2021.2343

[B33] Dubus Z. (2022). “High dose shock therapy with LSD and mescaline: the experiments of a French doctor on two homosexual adolescents in the 1960s,” in Queering Psychedelics, from oppression to libeation in psychedelic medicine, Belser, A., Cavnar, C., and Labate, B. (eds) (Santa Fe: Synergetic Press) p. 59–64.

[B34] Dubus Z. (2023). “Women, Mental Illness and Psychedelic Therapy in Postwar France,” in Expanding Mindscapes: a global history of psychedelics, Dyck, E., and Elcock, C. (eds). (Cambridge: MIT Press).

[B35] Dyck E. (2006). ‘Hitting highs at rock bottom': LSD treatment for alcoholism, 1950–1970. Soc. Hist. Med. 19, 313–329. 10.1093/shm/hkl039

[B36] Ey H. Igert C. Lairy G. (1959). État des recherches portant sur les hallucinogènes et les hallucinalytiques effectuées en 1958. Perpignan: Archives de Perpignan no. 380 in Fonds Ey (7S).

[B37] Gastaut H. Ferrer S. Castells C. Nicolle N. Luschnat K. (1953). Action de la diéthylamide de l'acide d-lysergique (LSD 25) sur les fonctions psychiques et l'électroencéphalogramme. Stereotact. Funct. Neurosurg. 13, 102–120. 10.1159/000105400 13060015

[B38] Guillemain H. (2020). Les effets secondaires de la technique. Patients et institutions psychiatriques au temps de l'électrochoc, de la psychochirurgie et des neuroleptiques retard (années 1940-1970). Revue d'histoire moderne contemporaine. 67, 72–98. 10.3917/rhmc.671.0072

[B39] Guyotat J. (1987). La formation des psychiatres en France. Raison présente. 83, 69–81. 10.3406/raipr.1987.2614

[B40] Hartogsohn I. (2017). Constructing drug effects: a history of set and setting. Drug Sci. Policy Law. 3, 1–17. 10.1177/205032451668332536321557

[B41] Heim R. (1924). Une exposition mycologique automnale à Gap. Quelques mots sur la comestibilité des champignons dans les hautes régions dauphinoises. Bulletin trimestriel de la Société mycologique de France Tome XL. p. 193–202.

[B42] Heim R. (1956). ETHNOMYCOLOGIE - Les champignons divinatoires utilisés dans les rites des Indiens Mazatèques, recueillis au cours de leur premier voyage au Mexique, en 1953, par Mme Valentina Pavlovna Wasson et M. R. Gordon Wasson. Comptes-rendus des séances de l'Académie des sciences. 242, 965–968.

[B43] Heim R. (1957). “Analyse de quelques expériences personnelles produites par l'ingestion des Agarics hallucinogènes du Mexique,” in Comptes rendus hebdomadaires des séances de l'Académie des sciences (Paris: Académie des sciences) p. 597–603. 13461421

[B44] Heim R. (1978). Les champignons toxiques et hallucinogènes. Paris: Boubée.

[B45] Heim R. Wasson R. G. Hofmann A. (1958). Les champignons hallucinogènes du Mexique : études ethnologiques, taxinomiques, biologiques, physiologiques et chimiques. Paris: Muséum National d'Histoire Naturelle.

[B46] Henckes N. (2008). “Réformer et soigner. L'émergence de la psychothérapie institutionnelle en France, 1944-1955,” in Psychiatries dans l'histoire (Caen: Presses universitaires de Caen) p. 277–288.

[B47] Jay M. (2019). Mescaline: A Global History of the First Psychedelic. New Haven: Yale University Press. 10.12987/978030024508031823070

[B48] Knaueur A. Maloney W. (1913). A Preliminary Note on the Psychic Action of Mescalin, with Special References to the Mechanism of Visual Hallucinations. J. Nerv. Mental Dis. 40, 425–443. 10.1097/00005053-191307000-00001

[B49] Lanter R. Weil J. Roth M. (1962). Note à propos de l'utilisation diagnostique et thérapeutique des drogues hallucinogènes (mescaline –LSD-25). Annales Médico-Psychologiques II. 244–253. 13928711

[B50] Laurentin E. Artières P. Fauvel A. Guillemain H. (2019). “Quelle histoire de la folie après Foucault? - Ép. 3/4,” in France Culture, La fabrique de l'histoire.

[B51] Leary T. (1961). Letter from Timothy Leary to Jean Delay, June 30, 1961. New York: Timothy Leary Archives, NYC Public Library.

[B52] Majerus B. (2019). A chemical revolution as seen from below. The “discovery” of neuroleptics in the Paris of the 1950s. Social Hist. Med. 32, 395–413. 10.1093/shm/hkx113

[B53] Mayer-Gross W. Stein S. (1926). Über einige Abänderungen der Sinnestätigkeit im Meskalinrausch. Zeitschrift für die Gesamte Neurologie und Psychiatrie. 101, 354–386. 10.1007/BF02878343

[B54] Michaux H. (1955). Letter from Henri Michaux to Roger Heim, July 1955. Paris: box no. 5, Roger Heim collection of the MNHN library.

[B55] Michaux H. (1960). La psilocybine (Expériences et autocritique). Revue de mycologie. 25, 52–68.

[B56] Missa J.-N. (2006). Naissance de la psychiatrie biologique: Histoire des traitements des maladies mentales au XXe siècle. Paris: PUF.

[B57] Nicolas-Charles P. J. (1958). Letter from P. J. Nicolas-Charles to Roger Heim dated August 20, 1958. Paris: box no. 5, Roger Heim collection of the MNHN library.

[B58] Olié J.-P. (1992). Histoire d'une découverte en psychiatrie: 40 ans de chimiothérapie neuroleptique. Paris: Doin.

[B59] Perrine D. (2001). Visions of the Night Western Medicine Meets Peyote 1887-1899. Heffter Rev. 2, 6–52.

[B60] Postel J. Coss P. (1956). La thérapeutique par la psychose induite (mescaline et chlorpromazine). Annales Médico-Psychologiques. 114, 254–282. 13340391

[B61] Quétin A.-M. (1960). La psilocybine en psychiatrie clinique et expérimentale. Paris: Thèse de médecine, Paris.

[B62] Robert R. (1962). Contribution à l'étude des manifestations neuro-psychiques induites par la psilocybine chez le sujet normal: à propos de 35 protocoles réalisés chez des peintres. Paris: Thèse de médecine, Paris.

[B63] Ropert M. (1957). La mescaline en psychiatrie clinique et expérimentale. Paris: Thèse de médecine, Paris.

[B64] Rouhier A. (1927). Le peyotl: la plante qui fait les yeux émerveillés. Paris: G. Doin.

[B65] Rouhier A. (1989). Le peyotl: la plante qui fait les yeux émerveillés. Paris: Guy Trédaniel.

[B66] Snelders S. Kaplan C. Pieters T. (2006). On cannabis, chloral hydrate, and career cycles of psychotropic drugs in medicine. Bull Hist Med. 80, 95–114. 10.1353/bhm.2006.0041 16549883

[B67] Stévenin L. Benoit J.-C. (1960). À propos de l'utilisation des substances dysleptiques en psychothérapie. Résultats favorables de séances répétées. Encéphale. XLIX, 428–434.

[B68] Sutter J. M. Pélicier Y. (1957). “Les substances hallucinogènes,” in Encyclopédie Médico-Chirurgicale - Psychiatrie (Paris) p. 1–10.

[B69] Thévenard P. (1964). Les champignons hallucinogènes du Mexique. Film documentaire scientifique. Fondation Singer-Polignac.13414201

[B70] Thuillier J. (1981). Les dix ans qui ont changé la folie. Paris: Robert Laffont.

[B71] Toscano Aguilar M. (1959). Étude Auto-Expérimentale Avec Deux Drogues Psychodysleptiques: L.S.D 25 et Psylocybine; Comparaison Des Résultats.

[B72] Verroust V. Zafar R. Spriggs M. J. (2021). Psilocybin in the treatment of anorexia nervosa: the English transition of a French 1959 case study. Annales Médico-psychologiques, revue psychiatrique. 777–81. 10.1016/j.amp.2021.08.004

[B73] Virel A. (1971). Approches psychophysiologiques de l'imagerie mentale. Bulletin de psychologie 24, 682–692.

[B74] Wasson R. G. (1955). Letter from Robert Gordon Wasson to Roger Heim, July 1955. Paris: box n°5, Roger Heim collection of the MNHN library.

[B75] Wasson V. P. Wasson R. G. (1957). Mushrooms, Russia and history. New York: Pantheon Books.

[B76] Weil J. (1965). Essai d'utilisation des psychodysleptiques dans le traitement des alcooliques en hôpital psychiatrique. Paris: Thèse de médecine, Paris.

[B77] Widlöcher D. (1957). Le diéthylamide de l'acide lysergique; étude de psycho-pathologie expérimentale. Paris: Thèse de médecine, Paris.

[B78] Wilhem M.-T. (1955). Intérêt de l'épreuve mescalinique dans les maladies mentales. Paris: Thèse de médecine, Paris.

